# Toward a Comprehensive Raman Analysis of Functional Cells With Structured Optical Traps

**DOI:** 10.1021/acs.analchem.6c00651

**Published:** 2026-04-21

**Authors:** Panchanil Sarmah, Vidya Rastapur, Ruchee Khanna, Aseefhali Bankapur

**Affiliations:** 1 Manipal Institute of Applied Physics, 76793 Manipal Academy of Higher Education, Manipal 576104, India; 2 Department of Pathology, Kasturba Medical College, Manipal, Manipal Academy of Higher Education, Manipal 576104, India

## Abstract

Optically trapping RBCs with conventional Gaussian laser beams in physiological media and studying their properties have long been of interest, as it can be beneficial for understanding RBC-related disorders and their manifestations. An ever-ignored fact of such studies is that the point excitation spot of a tightly focused Gaussian beam inevitably probes only a small part of the cell’s interior region, mostly the cytoplasm. More importantly, it omits key components of the cell membrane, thereby lagging behind information from one of the most crucial organs of RBCs. Recent donut- and line focus trapping using a focused vortex beam and an optical sheet, respectively, have proven capable of extracting information from cell membranes. However, the donut and line focus trapping are distinctly different from each other in probing RBCs, as the former is most exclusive, and the latter is most inclusive in excitation of the cell membrane. This study aims to establish a clear and distinctive capability of point, donut, and line focus trapping in deriving Raman spectroscopy signatures from RBCs. Principal component analysis highlighted these differences by classifying spectra based on dimensionally reduced characteristic spectral features. ANOVA identified the significance of the membrane contribution in shaping the Raman spectra obtained using donut and line focus trapping. Line focus trapping also revealed that the spectra are rich in information, particularly in the 1220–1290 cm^–1^ range, corresponding to the amide III band.

## Graphical abstract

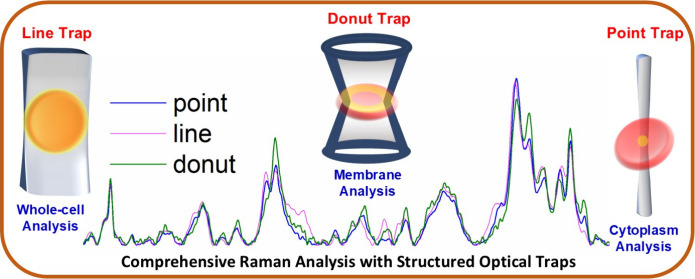

## Introduction

Understanding blood biochemistry is often considered a primary diagnostic parameter because it is linked to an organism’s physiological condition.^1^ RBCs, the major blood component, are critical in investigating many diseases and their progression.^2^ Red blood cells (RBCs) primarily transport oxygen from the lungs to different body organs. It regulates the systemic metabolism of nitric oxide (NO), redox metabolism, blood rheology, and viscosity.^3^ Disorders in the RBC population (polycythemia), morphology (anisocytosis, poikilocytosis, anisochromia/polychromasia^4^), and function (glucose-6-phosphate dehydrogenase (G6PD) deficiency, pyruvate kinase (PK) deficiency) are well-known. Traditional methods, such as complete blood count, peripheral blood smear observation, qualitative and quantitative assays, enzyme activity assays, and flow cytometry, among others, are used to understand and diagnose these abnormalities. Optical tweezers have been used as an unconventional method for RBC characterization, and their ability to study intact individual RBCs makes them a promising alternative.

Studying the physical properties of RBCs or hemorheology is an essential indicator of the pathogenesis of various disorders.^5^ Optical tweezers can be helpful in this regard, as they have been applied to study the rheological properties,^6^ biophysical properties,^7^ deformability,^8^ and RBC aggregation.^9–11^ Optical tweezers have been combined with other techniques such as Raman, microfluidics, and fluorescence,^12^ etc., which has greatly enhanced their applicability. Optical trapping of RBCs and obtaining Raman signatures have been of particular interest in the past two decades.^13^ Although success has been achieved in obtaining high-quality spectral signatures of RBCs, very few efforts have been made to explore alternative methods for trapping RBCs for comprehensive Raman probing. Such innovation is necessary because the traditionally used point spot of a Gaussian beam has a smaller focal volume, owing to the tight focusing required for trapping. A smaller portion of the RBC is exposed, and signals from inside the RBC, hence arriving from hemoglobin, dominate the Raman spectra.

Previous studies on disorders such as thalassemia and diabetes (type II) using a Gaussian beam have only revealed structural changes in hemoglobin.^14,15^ Changes in membrane lipids, proteins, cytoskeleton, and other components, as well as lipid–lipid, lipid–protein, protein–protein, hemoglobin-membrane, and hemoglobin-hemoglobin interactions, remain to be explored. The mechanical, morphological, and disruptive biochemical studies have already reported an altered membrane in thalassemia,^16^ detachment of the spectrin network from the lipid bilayer in Sickle cell anemia,^17^ and weakened cytoskeleton-lipid interaction in hereditary spherocytosis.^18^ Thus, developing a method for comprehensive biochemical analysis of intact, metabolically active RBCs with native biomolecules and classifying them based on these data is necessary.

Promising alternatives to conventional point-trap Raman tweezers use focused vortex beams or light sheets to create donut- or line-shaped optical traps, respectively, to study RBCs in an unconventional way.^19,20^ The donut traps have been successfully employed to study the membrane-level chemical changes in functional RBCs during elliptocytosis.^21^ However, the RBC spectra from the donut focus may lack contributions from the cell’s bulk. The inherently broad Rayleigh region of the line traps tends to excite a larger volume of RBCs, including their membranes. Additionally, the line-shaped focal image at the spectrometer slit, which is also rectangular, will increase the spectrometer throughput, resulting in increased spectral intensity and, thereby, reducing the excitation power and acquisition time, a vital requirement for photodamage-free measurements.

Thus, it is essential to evaluate the practical capabilities of each of the methods described above through a comparative study involving Raman measurements on a statistically significant number of RBCs. This study aims to conduct a comparative analysis of Raman spectral features acquired using point, donut, and line traps with an emphasis on developing a comprehensive biochemical analysis of RBCs.

## Experimental Methods

A home-built Raman tweezers setup, developed on a modified upright microscope platform with provisions for interchanging between point, donut, and line optical traps, had a resolution of ∼6.9 cm^–1^ and was used to acquire the Raman spectra of RBCs.^21^ A complete description of the conversion of the Gaussian beam into a vortex beam/light sheet and the associated advantages over the Gaussian beam can be found in our previous papers.^19,20^ In short, a 785 nm laser with a maximum output of 175 mW was used as an excitation source; 40× (0.75 NA) and 60× (1.00 NA) objectives were used for optical trapping and Raman signal collection, respectively. For the signal dispersion and detection, we employed an iHR320 spectrometer (Horiba Jobin Yvon) with a 1200 gr/mm grating blazed at 750 nm and a liquid-nitrogen-cooled CCD (Horiba Jobin Yvon). A schematic of the setup is shown in Figure S1.

Raman spectra from a total of 63 RBCs suspended in saline were recorded, of which 20 were using a point trap, 21 were using a donut trap, and 22 were using a line trap. The acquisition time was 60 s, and each spectrum is an average of two accumulations. RBCs from a single volunteer were used in this experiment to minimize donor-specific Raman spectral variations.^21^ The trapping/Raman excitation powers were maintained at 4.7, 17.4, and 21.8 mW for point, line, and donut traps, respectively. The power levels for each mode are determined based on their trapping potential and Raman throughput requirements, ensuring a fair spectral comparison. The power density was estimated to be 15 × 10^4^, 7.6 × 10^4^, and 9.8 × 10^4^ W/cm^2^ for the point, line, and donut traps, respectively. Studies conducted^22^ have shown that the rheological properties of RBCs do not change when trapped using 532 and 633 nm lasers at power levels below 100 mW for up to 5 min. Ref 23 showed that the power level at 1064 nm, known to cause significant RBC membrane damage, is ∼280 mW. Ref 24 also concluded that up to 20 min of 50 mW at 650 nm wavelength laser exposure did not cause any detectable changes in RBCs and did not denature membrane proteins. Thus, the power levels used are very low, comparatively, to cause any damage to the RBC membrane. To ensure the comparison is independent of the probed hemoglobin (Hb) amount, which varies with different excitations, we have normalized the spectra to the sum of intensities in the region 1503–1618 cm^–1^, which is sensitive to Hb concentration.^25,26^ The lower-wavenumber region was excluded from the comparison because the coverslip contributed most of it. The captured Raman spectra underwent standard preprocessing steps, including background removal and smoothing with the Savitzky–Golay filter (2nd-order polynomial with 9 points).

## Results and Discussion

We identified distinct spectral features in the spectra recorded by using the three trapping geometries. The average spectra were divided into four regions (i.e., regions I–IV) to facilitate spectral comparison and are shown in Figures 1 and 2. Region I (880–1180 cm^–1^) has signatures from protein skeletal stretching vibrations, viz., N–C_α_–C (α-helix) and N–C_α_–C (RCoil) at 896/899, 917, 931, and 955, 962, and 967 cm^–1^. Most of the protein C–N stretching vibrations are typically strong and appear at 1042, 1093, 1113, 1118, 1124, and 1130 cm^–1^. These vibrations overlap with those arising from lipids’ (i) headgroups, viz., at 889/893, and 906 cm^–1^; (ii) C–C stretching at 1056, 1063, 1066, 1081, 1089, and 1130 cm^–1^; and (iii) deformation vibrations [δ­(CH)] at 981, 1028, 1035, and 1040 cm^–1^. Contributions from individual amino acids appear at 885/888 [tryptophan: C_ε3_-C_ζ3_-C_η_], 902 [lysine: C_γ_-C_δ_], 931 [glutamic acid: γ­(OH)], 991 [proline: ring str], 1000 [phenylalanine: C_γ_-C_δ1_], 1012 [tryptophan: C_ζ3_-C_η_ ], 1028 [phenylalanine: C_ε1_-C_ζ_, lipid: δ­(CH)], 1046/1051 [arginine: C_γ_-twist], 1066 [lysine: C_δ_-C_ε_], 1071 [histidine: C_α_-C_β_], 1073 [glutamic acid: ν­(CN), ν­(CC)], 1079 [tryptophan: N_ε1_-C_δ1_-C_γ_, lysine: C_γ_-C_δ_], 1088 [histidine: C_δ_-N_ε_, arginine: C_ζ_N_η1_H_2_], 1161 [protein: CH_3_ rock], 1167 [arginine: C_β_-C_α_-H_α_], and 1179 [arginine: C_ζ_N_η2_H_2_]. The heme peaks appear at 942 [ν_32_], 974 [ν_46_], 1093 [δ­(=C_b_H_2_)_as_], 1118 [ν_5_], 1124 [ν_5_], 1151 [ν_44_], and 1171 [ν_30_] cm^–1^. Some contributions from carbohydrates can be seen at 885/888 [Gal-amine], 896/899 [sialic acid], 913 [glucose], 1051 [galactose, glucose], 1073 [galactose, sialic acid], 1088 [mannose], 1113 [fucose, sialic acid], 1130 [fucose], 1142 [galactose, sialic acid], 1148 [glucose], and 1157 [galactose, fucose, Gal-amine] cm^–1^.

**Figure 1:**
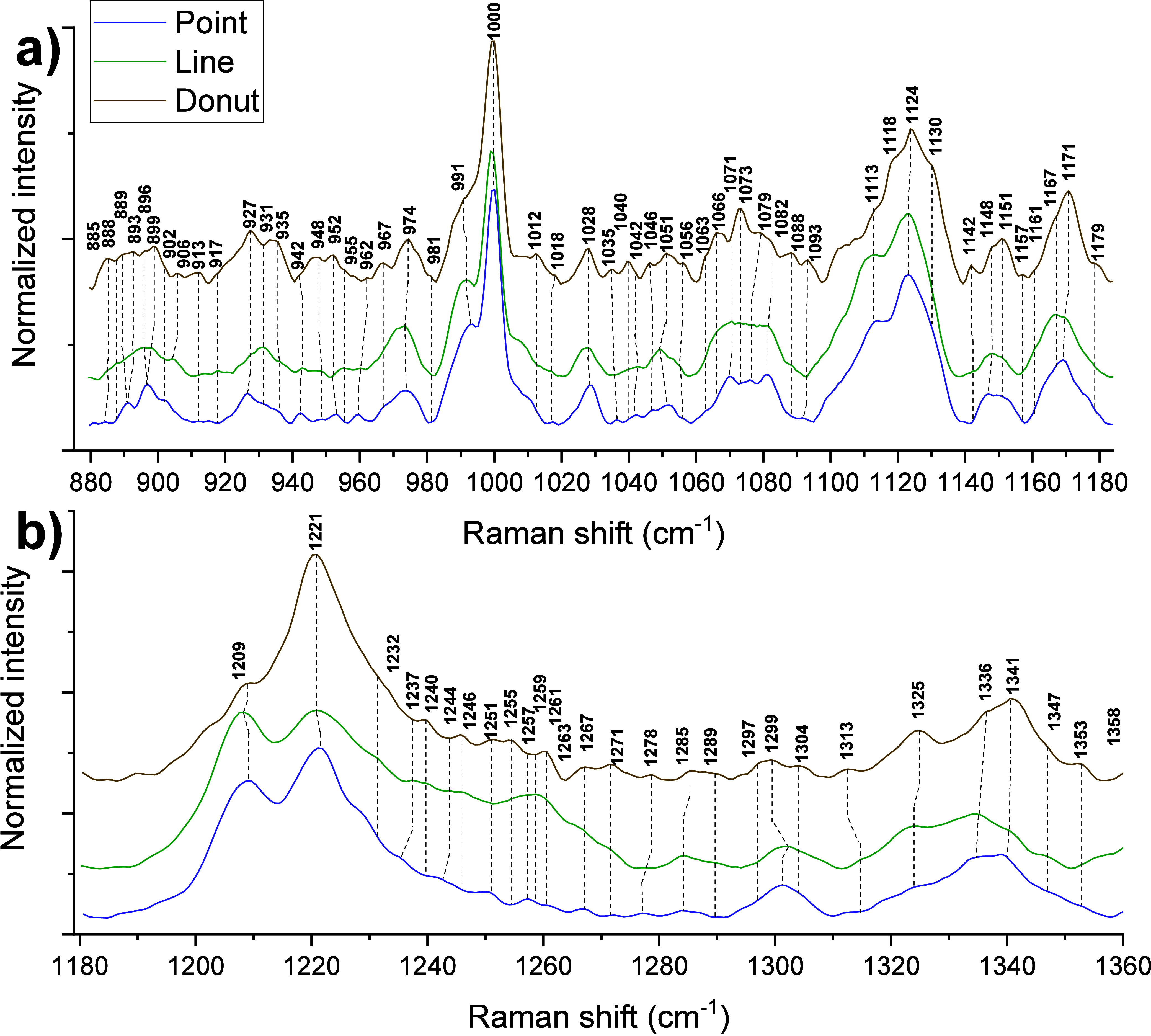
Raman spectra recorded using three trapping geometries: (a) region I (880–1180 cm^–1^), and (b) region II (1180–1360 cm^–1^).

**Figure 2:**
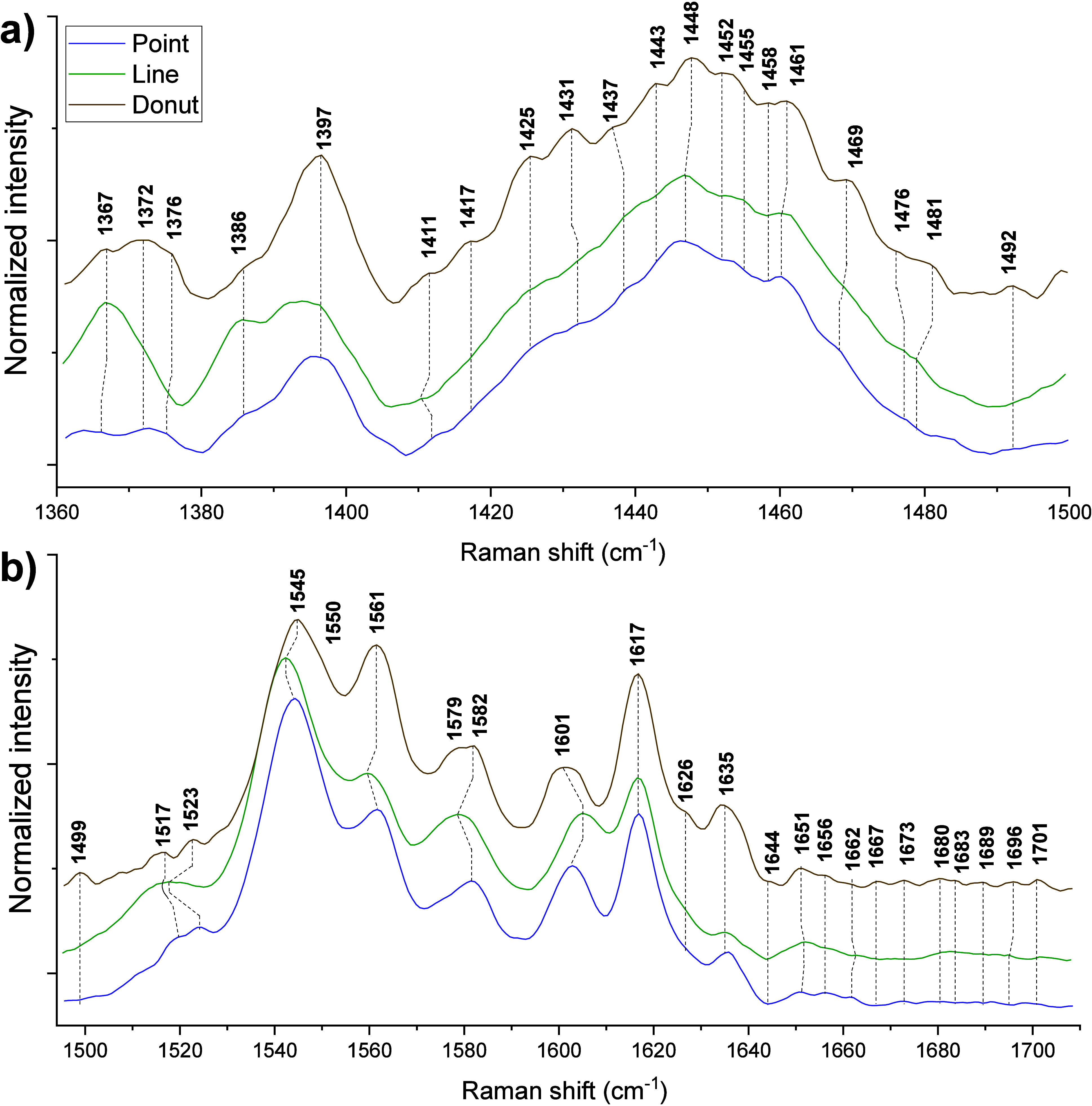
Raman spectra recorded using three trapping geometries: (a) region III (1361–1495 cm^–1^); (b) region IV (1495–1708 cm^–1^).

Region II (1180–1360cm^–1^) comprises strong heme vibrations at 1209 [ν_5+_ν_18_], 1221 [ν_13_], 1285 [methlyene wag], 1299 [ν_21_], 1336 [ν_41_], and 1341 [ν_41_] cm^–1^. It also has amide III band (1229–1278 cm^–1^) bands and other amino acids, lipid bands, viz., 1232 [amide III (β-sheet)], 1237 [amide III (β-sheet), histidine: C_β_-twist], 1240 [amide III (β-sheet), 1244 [amide III (unordered), 1246 [amide III (unordered), lipid: PO_2_^–^ antisym str], 1251 [amide III (unordered), tyrosine], 1255/1257 [amide III (β-turn), tryptophan: C_ε2_-N_ε1_], 1259 [amide III (β-turn), tryptophan], 1261 [amide III (β-turn), tryptophan], 1263 [amide III (β-turn), 1267 [amide III (β-turn), tyrosine: C_ζ_-O-H; histidine: N_δ_-C_ε_-H; phosphatidylcholine, phosphatidlyserine, phosphatidylinositol: δ­(=CH)], 1271 [amide III (α-helix/β-turn), fucose], 1279 [Amide III (α-helix/β-turn), Tryptophan: C_ε2_-C_ζ2_-H], 1289 [α-helix], 1297 [phosphatidylserine, phosphatidylethanolamine, sphingomyelin], 1304 [α-helix; phosphatidylcholine, phosphatidylinositol: τ­(CH_2_)], 1313 [glutamic acid: ω­(CH_2_), protein: δ­(C_α_–H)], 1325 [tryptophan: C_β_-rock], 1336 [protein: δ­(C_a_–H)], 1341 [ν_41_], 1347/1353 [tryptophan: C_δ2_-C_γ_], and 1358 [Hist, Lys, and Arg] cm^–1^.

Region III (1361–1495 cm^–1^) consists of some heme-specific bands 1367 [ν_4_], 1372 [ν_4_], 1376 [ν_4_], 1386 [ν_12_], and 1397 [ν_40_] cm^–1^. The remaining bands primarily consist of CH2/CH3 deformations from proteins and lipids, with a few additional contributions. Bands appear at 1411 [ν_29_ ], 1417 [lysine: C_δ_-rock, arginine: rock C_β_-rock], 1425 [lipid: β­(CH_2_), ν_28_], 1431 [lipid: β­(CH_2_)], 1437 [tryptophan: C_δ2_-C_ε3_-H], 1443 [histidine: C_β_-rock, lipid: α­(CH_2_/CH_3_)], 1447 [protein: δ­(CH_2_/CH_3_)], 1452 [protein: δ­(CH_2_/CH_3_)], 1455 [lipid: β­(CH_2_/CH_3_)], 1458 [glutamic acid: δ­(CH_2_), Man, Glu, Sia], 1461 [tryptophan: C_β_-bend], 1469 [lysine: C_ε_-bend, C_δ_-bend, and arginine: C_δ_-bend; C_γ_-bend], 1476 [protein: δ­(CH_2_/CH_3_)], 1481 [ν_3_, protein: δ­(CH_2_/CH_3_)], and 1492 [histidine: C_ε_-N_ε_] cm^–1^.

Region IV (1495–1708 cm^–1^) consists of two subregions: the spin-state marker region (1500–1637 cm^–1^) and amide I (1644–1708 cm^–1^). The spin state marker region includes peaks at 1499 [ν_39_], 1517 [ν_38,_ tyrosine: C_δ1_-C_ε1_-H], 1523 [ν_38_], 1545 [ν_11_], 1550 [tryptophan: C_γ_-C_δ1_, amide II], 1561 [ν_2_], 1579 [tryptophan], 1582 [ν_37_], 1601 [ν_10_], 1617 [ν­(C_a_=C_b_)_vinyl_], 1626 [Ace-Glu], and 1635 [ν_10_] cm^–1^. The amide I region consists of 1644 [histidine], 1651 [amide I (α-helix)], and 1656 [lipid: C=C str], 1662 [amide I (unordered)], 1667 [amide I (unordered)], 1673 [amide I (β-sheet), cholesterol: C=C str], 1680 [amide I (β-sheet)], 1683 [amide I (β-sheet)], 1689 [amide I (β-turn)], 1697 [amide I (β-turn)], 1701 [amide I (β-turn)].

Next, we employed PCA to identify any spectral changes that may have led to classification among the spectra recorded using the three trapping geometries. The PCA score plot of the first two principal components (PCs) in Figure 3 showed three groups clustering the spectra collected using point, donut, and line traps. The first two PCs explain approximately 84.6% of the variance in the data, with 61.3% contributed by PC1 and the remaining 23.3% contributed by PC2. Table S1 lists the spectral features present and picked up by different PC loadings in point, donut, and line traps, with columns 2–4 listing Raman frequencies observed in the individual spectra. Columns 5 and 6 show the peaks detected by PC2, which are also present in the difference spectra of the donut and line traps from the point trap, respectively. Column 7 shows the PC1 loadings. Columns 8–11 are the spectral assignments associated with the peaks.

**Figure 3:**
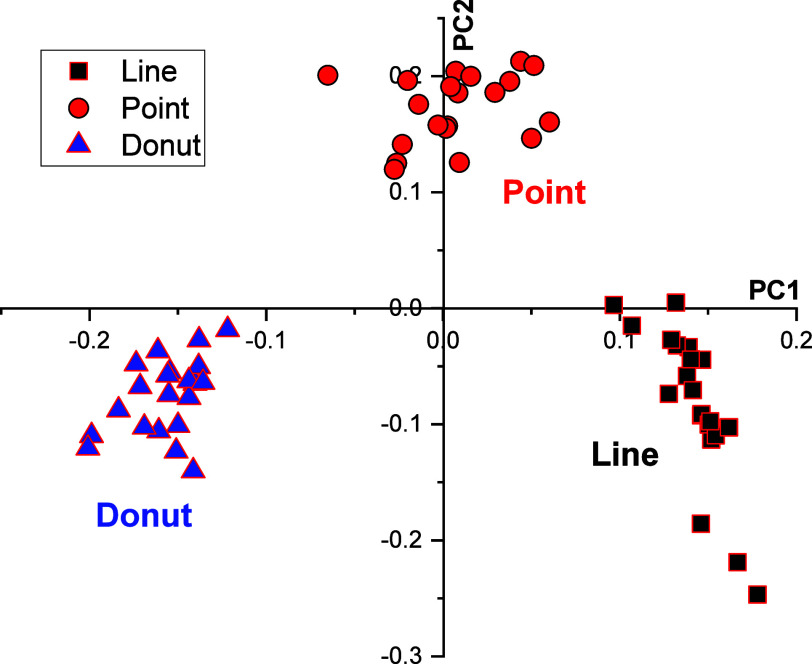
PCA scoreplot: PC1 vs PC2.

PC1 appears to correlate with differences between spectra collected with the donut and the line trap. Thus, we relate the difference spectrum, calculated by subtracting the average spectrum of the donut trap from that of the line trap, to the PC1 loadings. This was indeed the case, as evidenced by the PC1 loadings, which coincided with the said difference spectrum in Figure 4, indicating the role of spectral variations in the classification.

**Figure 4:**
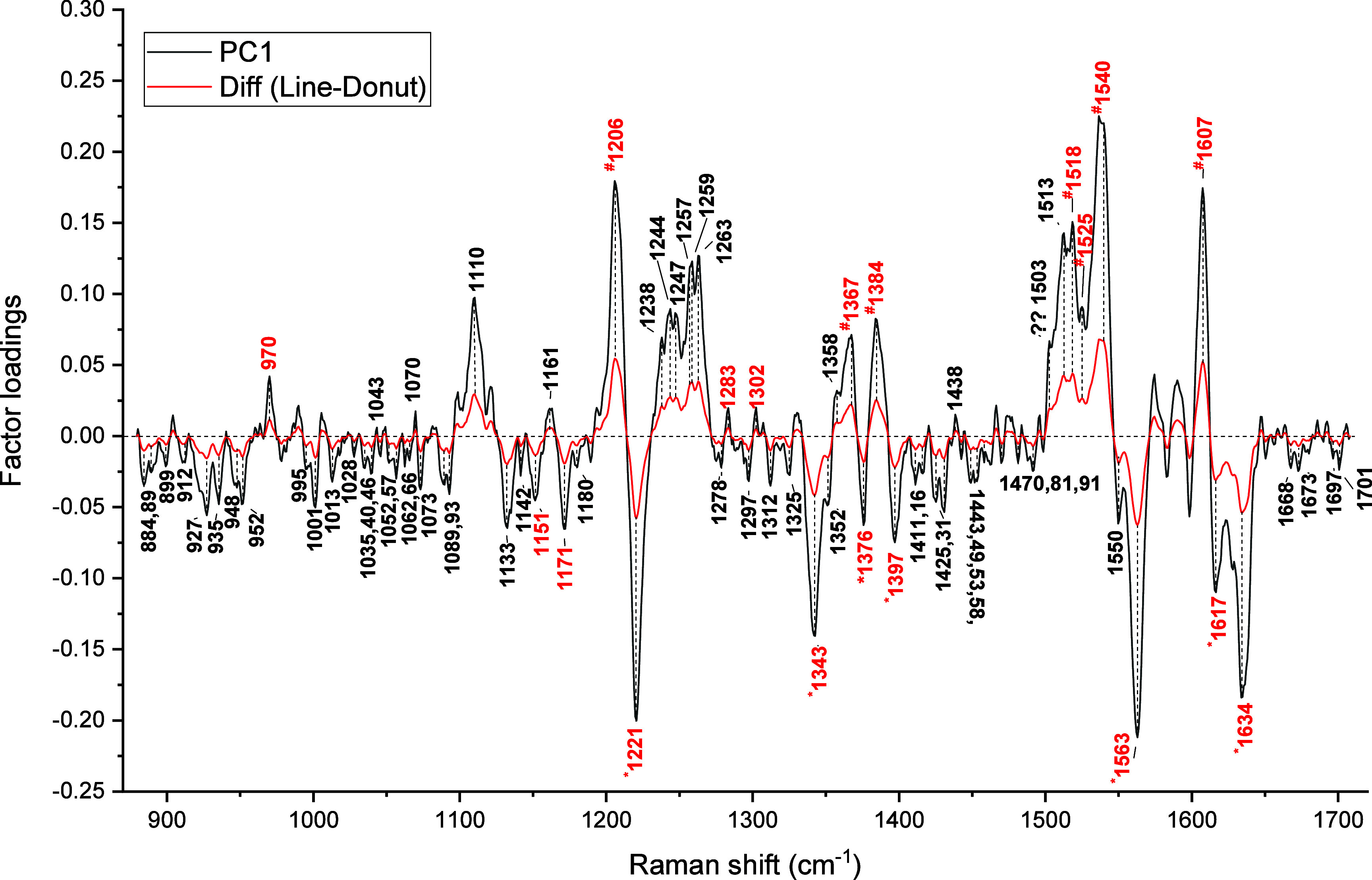
Comparison of the difference spectrum between line and donut trap RBCs with PC1 loadings (the peaks marked in red are Hb contributions).

The highest contributions in the PC1 loadings are mostly those arising from hemoglobin’s oxygenation state, viz., ^#^1206 [ν_5_+ ν_18_], *1221 [ν_13_], *1343 [ν_41_], ^#^1367 [ν_4_], *1376 [ν_4_], ^#^1384 [ν_12_], *1397 [ν_40_], ^#^1518/1525 [ν_38_], ^#^1540 [ν_11_], *1563 [ν_2_], ^#^1607 [ν_10_], *1617 [ν­(C_a_=C_b_)_vinyl_], and *1634 [ν_10_] cm^–1^ where * means oxygenation and ^#^ means deoxygenation marker. The spectra recorded using a line trap have acquired signal mainly from Hb molecules in the deoxygenated state. This is expected, as the broad Rayleigh length of the line trap covers a larger part of the RBC and thus involves signal from Hb molecules across most of the cell volume. Therefore, the line-trap excitation will reveal the overall oxidation status of RBCs. Other hemoglobin-related peaks that showed a change are 970 [ν­(C_c_-C_d_)], 1093 [δ­(=C_b_H_2_)_as_], 1151 [ν_44_], 1171 [ν_30_], 1283 [Methylene wag], 1302 [ν_21_], 1425 [ν_28_], and 1481 [ν_3_] cm^–1^.

PC1 also revealed that the peaks at 1043 [tyrosine, protein: C–N str], 1070 [histidine], 1110 [protein: C–N, sphingomyelin: C–C str, fucose, sialic acid], 1161 [protein: CH_3_ rock], 1238 [histidine, amide III (β-sheet), Gal-amine], 1244 [amide III (unordered str)], 1247 [amide III (unordered str), lipid: PO_2_^–^ antisym str], 1257,1259 [amide III (β-turn), tryptophan: C_ε2_-N_ε1_], 1263 [amide III (β-turn), 1358 [histidine, lysine, arginine], and 1438 [tryptophan: C_δ2_-C_ε3_-H] are relatively stronger in line-trap Raman spectra. Of these, the 1238, 1244, 1247, 1257, 1259, and 1263 cm^–1^ bands contribute significantly to PC1.

Other peaks from regions I, II, III and IV that showed a relative increase in the donut trap spectra are 884 [tryptophan: C_ε3_-C_ζ3_-C_η_], 889 [phosphatidylcholine, phosphatidylinositol], 899 [protein: sk: N–C_α_–C (α-helix), sialic acid], 912 [glucose], 927 [p: sk: N–C_α_–C (α-helix)], 935 [?], 948 [?], 952 [?], 995 [proline: ring str], 1001 [phenylalanine: C_γ_-C_δ1_], 1013 [tryptophan: C_ζ3_-C_η_], 1028 [phenylalanine: C_ε1_-C_ζ_, lipid: δ­(CH)], 1035 [lipid: δ­(CH)], 1040 [lipid: δ­(CH)], 1046 [arginine], 1052 [arginine: C_γ_-twist, galactose, glucose], 1057 [phosphatidylserine: C–C str], 1062 [sphingomyelin: C–C str], 1066 [lysine: C_δ_-C_ε_, phosphatidylcholine, phosphatidylethanolamine, phosphatidylinositol, sphingomyelin], 1073 [glutamic acid: ν­(CN), ν­(CC), galactose, sialic acid], 1089 [histidine: C_δ_-N_ε_, arginine: C_ζ_N_η1_H_2_; Phosphatidylcholine, Phosphatidylserine, and Phosphatidylethanolamine: C–C str, Mannose], 1093 [Phosphatidylinositol: P–O str], 1133 [protein: C–N; Sphingomyelin, Cholesterol: C–C str, Fucose], 1142 [galactose, sialic acid], 1180 [arginine: C_ζ_N_η2_H_2_], 1278 [amide III (α-helix/β-turn), tryptophan: C_ε2_-C_ζ2_-H], 1297 [phosphatidylserine, phosphatidylethanolamine, sphingomyelin], 1312 [glutamic acid: ω­(CH_2_), protein: δ­(C_α_–H)], 1325 [tryptophan: C_β_-rock], 1352 [tryptophan: C_δ2_-C_γ_], 1416 [lysine: C_δ_-rock, arginine: C_β_-rock], 1425 [lipid: β­(CH_2_)], 1431 [lipid: β­(CH_2_)], 1443 [histidine: C_β_-rock, lipid: α­(CH_2_/CH_3_)], 1449 [protein: δ­(CH_2_/CH_3_)], 1453 [protein: δ­(CH_2_/CH_3_)], 1458 [glutamic acid: δ­(CH_2_), mannose, glucose, sialic acid], 1470 [lysine, arginine, Ace-Glu], 1481 [protein: δ­(CH_2_/CH_3_)], 1491 [histidine], 1513 [tyrosine], 1550 [tryptophan: C_γ_-C_δ1_], 1668 [amide I (unord stru)], 1673 [amide I (β-sheet), cholesterol: C=C str], 1697 [amide I (β-turn)], and 1701 [amide I (β-turn)] cm^–1^.

From these changes, we can speculate that the donut focus excites a better portion of the lipid bilayer and some of the transmembrane proteins within it. This is supported by an increase of the peaks in the CH_2_/CH_3_ deformation region common to lipids and proteins: 1425, 1431, 1443, 1449, 1453, 1458, and 1481 cm^–1^ along with an increase in peaks 884, 889, 927, 1035, 1040, 1057, 1062, 1066, 1073, 1089, and 1133 cm^–1^ which belong to C–C skeletal and C–N vibrations. In contrast, the line beam is more sensitive to the amide III region (1238, 1244, 1247, 1257, 1259, and 1263 cm^–1^), indicating its usefulness in identifying protein conformational changes.

The changes detected by PC2 are quite interesting, as they relate to how spectra collected with donut and line traps differ from those collected with point traps. Since the donut and line trap are inseparable along the PC2 axis, the PC2 component also picked up similarities between them. The similarity may be due to the contribution from membrane components, as donut trap spectra have already been shown to have an increased contribution from the membranes.^19^

Individual comparisons of line- and donut-focus excited spectra with Gaussian-beam excited spectra will provide a better idea of their membrane contributions. Figures 5 and 6 compare PC2 loadings with the difference spectra calculated by subtracting the average spectra of the donut and line trap, respectively, from the point-trap spectrum. The PC2 has selected mostly the same spectral differences in both cases, with a few exceptions specific to donut or line traps. The common spectral differences include intensity variations at 887 [tryptophan: C_ε3_-C_ζ3_-C_η_], 893 [phosphatidylcholine, phosphatidylinositol], 902 [lysine: C_γ_-C_δ_], 907 [phosphatidylcholine, phosphatidylserine], 919/918 [protein: N–C_α_–C (α-helix)], 955 [protein: N–C_α_–C (RCoil)], 964 [protein: N–C_α_–C (Rcoil)], 973 [ν­(C_c_-C_d_)], 980 [lipid: δ­(CH)], 1015 [tryptophan: C_ζ3_-Cη], 1029 [phenylalanine: C_ε1_-C_ζ_, lipid: δ­(CH)], 1034 [lipid: δ­(CH)], 1040/1039 [lipid: δ­(CH)], 1055 [phosphatidylserine: C–C str], 1066 [lysine: C_δ_-C_ε_; phosphatidylcholine, phosphatidylethanolamine, phosphatidylinositol, sphingomyelin], 1073 [glutamic acid: ν­(CN), ν­(CC), galactose, sialic acid], 1079/1076 [tryptophan: N_ε1_-C_δ1_-C_γ_, lysine: C_γ_-C_δ_], 1089 [histidine: C_δ_-N_ε_, arginine: C_ζ_N_η1_H_2_; phosphatidylcholine, phosphatidylserine, phosphatidylethanolamine: C–C str, Man], 1094 [phosphatidylinositol: P–O str, Ace-Glu, Gal-amine, sialic acid], 1118 [protein: C–N], 1124 [protein: C–N], 1142 [galactose, sialic acid], 1166 [arginine: C_β_-C_α_-H_α_], 1172 [ν_30_], 1232 [amide III (β-sheet)], 1240 [amide III (β-sheet)], 1246 [amide III (unordered str), lipid: PO_2_^–^ antisym str, galactose], 1255 [amide III (unordered str), tryptophan], 1260/1259 [amide III (β-turn)], 1271 [amide III (α-helix/β-turn)], 1286/1285 [methylene wag], 1289 [α-helix], 1296 [phosphatidylserine, phosphatidylethanolamine, sphingomyelin], 1301 [ν_21_], 1324 [tryptophan: C_β_-rock, galactose, fucose, glucose], 1367 [ν_4_], 1416 [lysine: C_δ_-rock, arginine: C_β_-rock], 1448/1447 [p:δ­(CH_2_/CH_3_), fucose], 1431 [lipid: β­(CH_2_)], 1437 [tryptophan: C_δ2_-C_ε3_-H], 1452 [protein: δ­(CH_2_/CH_3_)], 1458 [glutamic acid: δ­(CH_2_), Man, Glu, Sia], 1463/1465 [tryptophan: C_β_-bend], 1471 [lysine: C_ε_-bend, C_δ_-bend, arginine: C_δ_-bend; C_γ_-bend, Ace-Glu], 1481/1479 [protein: δ­(CH_2_/CH_3_), ν_3_], 1519/1524 [ν_38_], 1546 [ν_11_], 1559/1565 [ν_2_], 1577 [ν_37_], 1602 [ν_10_], 1620 [ν­(C_a_=C_b_)_vinyl_], 1653 [amide I (α-helix)], 1683 [amide I (β-sheet)], 1690/1689 [amide I (β-turn)], 1696 [amide I (β-turn)], and 1701/1703 [amide I (β-turn)] cm^–1^. Most of these Raman frequencies originate from lipids, proteins, and carbohydrates. They are expected to have a higher contribution from molecules of noncytoplasmic origin, as they are relatively weak in point-trap spectra.

**Figure 5:**
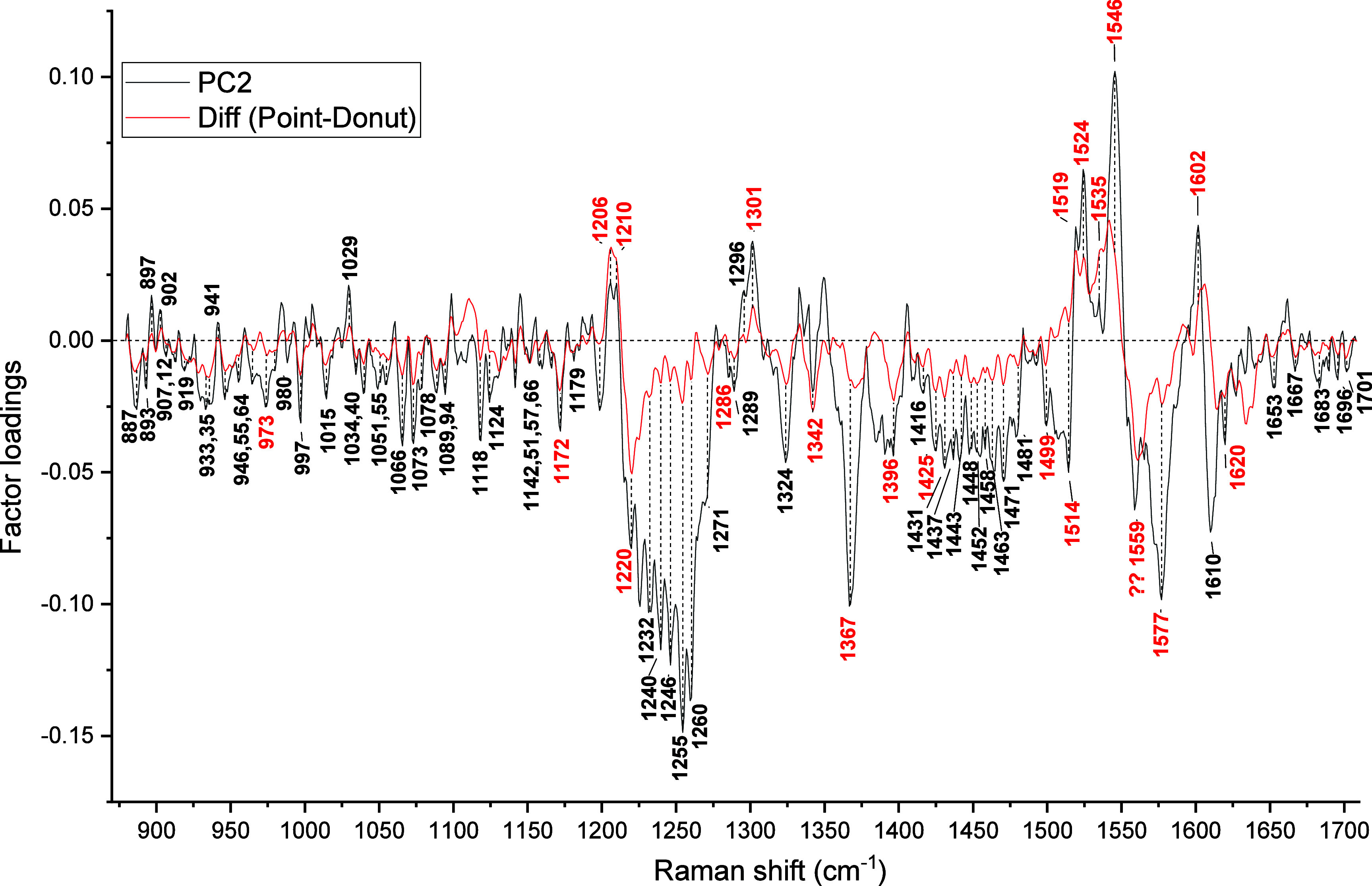
Comparison of difference spectra between point and donut trap RBCs with PC2 loadings (the peaks marked in red are Hb contributions).

**Figure 6:**
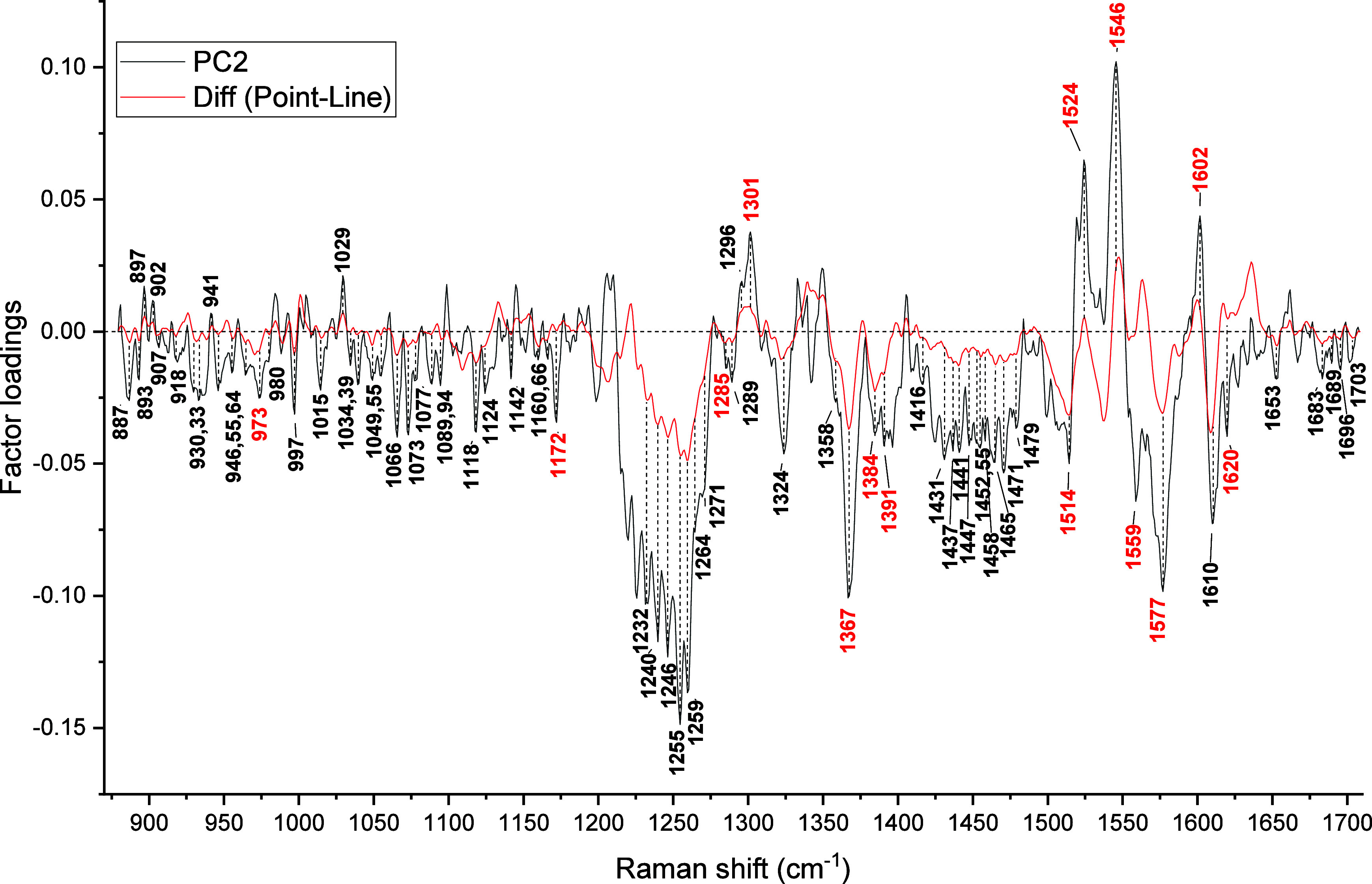
Comparison of difference spectra between point and line trap RBCs with PC2 loadings (the peaks marked in red are Hb contributions).

Furthermore, descriptive statistics and significance tests are used to assess the significance of the observed spectral changes. Figure 7 compares the central tendency and variability of some RBC membrane components across three trapping modes. The box plots show that, for all membrane components, the donut- and line-trapped spectra exhibited a significant (*p* < 0.001) contribution compared to the point-trapped spectra. The p-values for all of the Raman peaks picked by PC2 are given in Table S2, and it is obvious that most of the peaks showed a significant contribution in the donut- and line-trapped spectra. Thus, both the donut and line-trap excitations of RBCs have increased contributions from membrane components.

**Figure 7:**
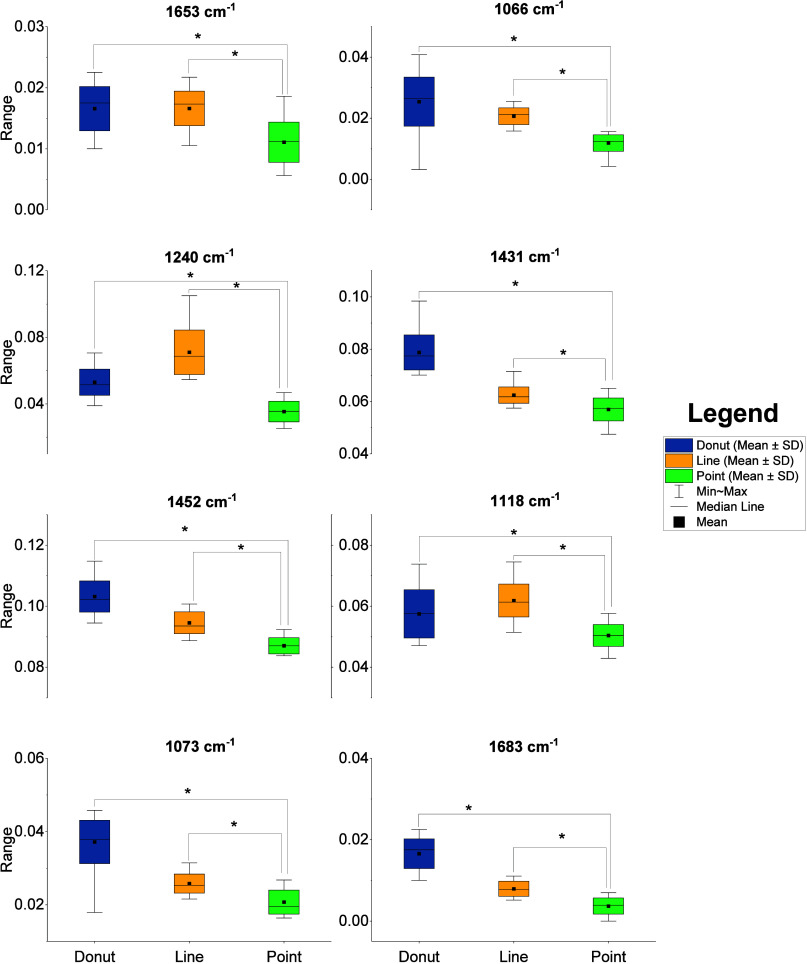
Descriptive statistics of some of the membrane components. Box plot: median, mean, whiskers: max–min, *p*-values: Welch’s ANOVA, *p < 0.001.

However, some Raman frequencies are not common to donut and line excitations. Some spectral features in PC2 match only with the difference spectrum calculated by subtracting the donut trap spectrum from the point-trap spectrum. They are 912 [glucose], 1151 [ν_44_], 1157 [galactose, fucose, Gal-amine], and 1179 [arginine: C_ζ_N_η2_H_2_], 1220 [ν_13_], 1342 [ν_41_], 1396 [ν_40_], 1443 [histidine: C_β_-rock, lipid: α­(CH_2_/CH_3_)], 1493 [histidine: C_ε_-N_ε_], 1499 [ν_39_], 1514 [tyrosine: C_δ1_-C_ε1_-H], 1667 [amide I (unordered)]. Of these peaks, 1151, 1220, 1342, 1396, and 1499 cm^–1^ belong to hemoglobin and have no membrane contribution. Only 912, 1443, and 1667 cm^–1^ are prominent in the spectra collected using the donut trap compared to the rest of the peaks. In the case of line trap peaks 930 [protein: N–C_α_–C (α-helix), glutamic acid: (γ­(OH))], 1160 [CH_3_ rock], 1259 [amide III (β-turn), tryptophan], 1264 [amide III (β-turn)], 1358 [histidine, lysine, arginine], 1441 [histidine, lipid: α­(CH_2_/CH_3_)], 1455 [lipid: β­(CH_2_/CH_3_)] have been picked up PC2 which are not present in the spectra collected using donut trap. Only peaks at 930 and 1259 cm^–1^ are prominent in the line-trap spectra collected. Thus, only a few exceptions, viz., 912, 930, 1259, 1443, and 1667 cm^–1^, are considerable, indicating that PC2 shows the most similar membrane components.

Typically, the Raman spectral features of hemoglobin are sensitive to photodamage. Among the spectral changes, the increase in Raman frequency at 970 cm^–1^ and the increase in the 1367-to-1397 cm^–1^ peak intensity ratio are known to represent the level of photothermal heme aggregation in RBCs. Similarly, the intensity increases at 1518 and 1607 cm^–1^, the decrease at 1634 cm^–1^, and the increases in the 1206-to-1221 and 1540-to-1563 cm^–1^ peak intensity ratios are known to identify photooxidation in heme, resembling the spectral changes associated with the oxy-deoxy transition. To determine the level of photodamage across the three modes of optical trapping, we performed descriptive statistics on the peak intensities at 970, 1518, 1607, and 1635 cm^–1^, as well as the peak intensity ratios of 1206/1221, 1367/1397, and 1540/1563, as identified by PC1. The data for heme aggregation markers are shown in Figure 8a,b, and those for the photooxidation marker are shown in Figure 8c–g.

**Figure 8:**
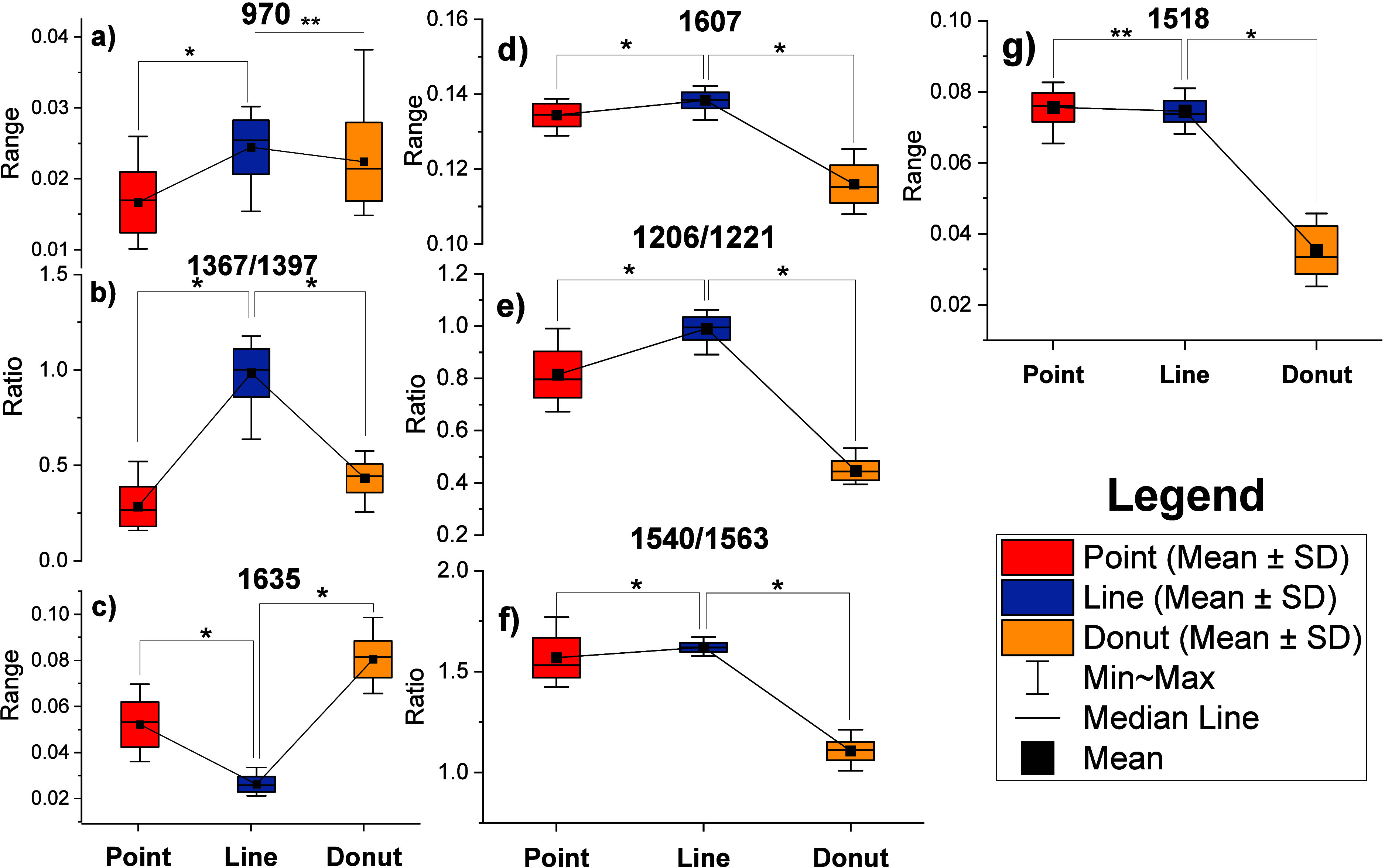
Descriptive statistics on heme aggregation (a, b) and photooxidation peaks (c–g). Box plot: median, mean, whiskers: max–min, *p*-values: Welch’s ANOVA, *p < 0.05, **p > 0.05.

It is clear from Figure 8a,b that, among the three trapping modes, photothermal heme aggregation is the least in spectra obtained using point trapping and is relatively higher in line-trap spectra. Although the power density in the point trap is twice that in the line-trap, heme aggregation signatures are less pronounced in the point trap, which may be due to the significant difference in excitation volume between the two traps. The whole-cell coverage by the line trap includes most heme molecules, whereas the point trap does not. Similarly, the photooxidation is least in the spectra obtained using the donut trap and is relatively higher in the line-trap spectra, as shown in Figure 8c–g. This could be because, in the case of donut trapping, the cells are oriented face-on along the edges, resulting in fewer heme molecules being excited. Moreover, the heme aggregation and photooxidation levels observed in this study are significantly lower and within an acceptable range, as previously studied.^21^

To further evaluate the evolution of heme aggregation and photoxidation in the line trap, we recorded Raman spectra of trapped RBCs at acquisition times ranging from 10 to 60 s, at 10 s intervals. Figure 9 shows the variation in the marker peaks and ratios. Five RBC spectra have been obtained for each acquisition time. Most of the makers showed slight variance, except for the 1367-to-1397 cm^–1^ and 1206-to-1221 cm^–1^ peak intensity ratios and the 1635 cm^–1^ peak intensity. These results suggest that reducing the acquisition time to 30–40 s may benefit line-trap Raman acquisition, albeit at the expense of throughput. Moreover, these spectral variations are well understood and contribute minimally, so they are accounted for during the analysis.

**Figure 9:**
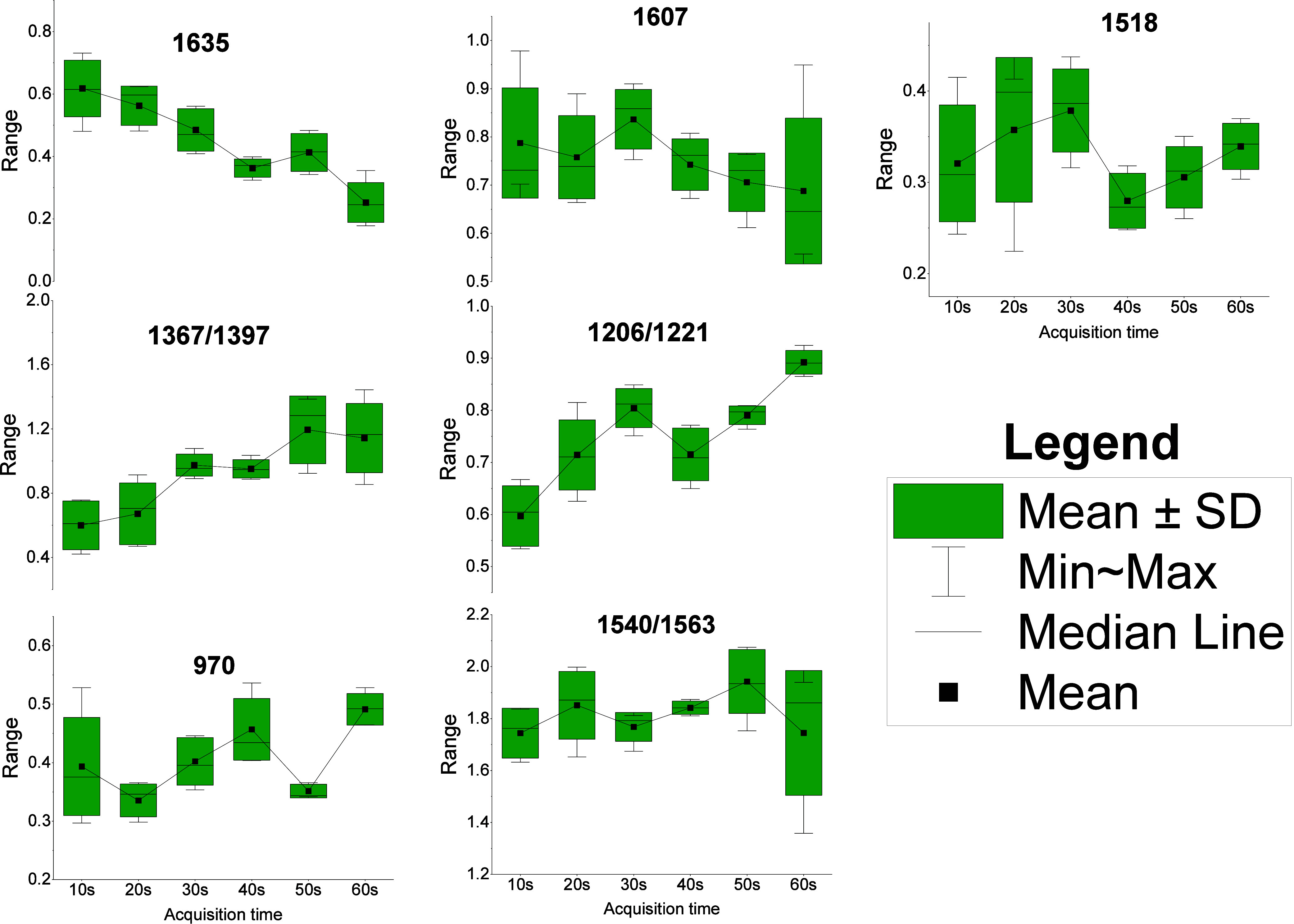
Variations of heme aggregation and photodamage markers for different acquisition times. Box plot: median, mean, whiskers: max–min.

## Conclusions

The study successfully compared the biochemical information obtained by Raman spectroscopy of single, functionally active RBCs, optically trapped with point, donut, and line focus. Principal component analysis (PCA) could extract the discriminating spectral features from the multidimensional Raman data, forming distinct groups for each of the three excitations in the PCA score space. Among the first two principal components (PCs), PC2 played a prominent role, representing features corresponding to RBC membrane components that separate the donut- and line focus excited spectra from the point-spot excited spectra. Concurrently, PC1 accounted for the distinguishing features of hemoglobin and the amide III region in spectra obtained with donut- and line focus excitation. Furthermore, comparing spectra recorded with the donut and line traps indicated that the donut trap remains the most effective method for probing the membranes of functionally active cells. Owing to the difficulty in forming a stable donut trap for nondiscoid cells, a line trap is an immediate substitute for the greater applicability to the membrane studies of morphologically unsymmetric, spherical, and other cells with different morphologies that are difficult to trap using a donut trap. The line trap is also more sensitive in the amide III region, which is beneficial for identifying protein conformational changes. On a broader scale, a Raman system that easily switches between these three modes can provide comprehensive data at the single functional cell level.

## Supplementary Material



## Notes

Schematic of the experimental setup used in the study, a table comparing spectral features of each trap mode with principal components, and another table listing *p*-values for descriptive statistics of membrane-specific Raman frequencies (PDF)

## References

[1] Avsievich T., Zhu R., Popov A., Bykov A., Meglinski I. (2020). The advancement
of blood cell research by optical tweezers. Reviews in Physics.

[2] Barbalato, L. ; Pillarisetty, L. S. Histology, Red Blood Cell; StatPearls Publishing. 14 November 2022. https://www.statpearls.com/point-of-care/28286. 30969524

[3] Kuhn V., Diederich L., Keller T. C. S., Kramer C. M., Lückstädt W., Panknin C., Suvorava T., Isakson B. E., Kelm M., Cortese-Krott M. M. (2017). Red Blood Cell Function and Dysfunction: Redox Regulation,
Nitric Oxide Metabolism, Anemia. Antioxidants
& Redox Signaling.

[4] Adewoyin, A. S. ; Adeyemi, O. ; Davies, N. O. ; Abiola Ogbenna, A. , Erythrocyte Morphology and Its Disorders. In Erythrocyte, Tombak, A. , Ed.; IntechOpen: Rijeka, 2019. DOI: 10.5772/intechopen.86112.

[5] Alexy T., Detterich J., Connes P., Toth K., Nader E., Kenyeres P., Arriola-Montenegro J., Ulker P., Simmonds M. J. (2022). Physical
Properties of Blood and their Relationship to Clinical Conditions. Frontiers in Physiology.

[6] Liu Y., Rao H., Zhang H., Wang M., Wu Y., Wu Y., Han C., Yan C., Zhang L., Chen W., Wang J. (2024). Efficient
characterization of red blood cell rheological properties using a
multichannel microfluidic chip and optical tweezers. Materials Today Advances.

[7] Tognato R., Bronte Ciriza D., Maragò O. M., Jones P. H. (2023). Modelling red blood
cell optical trapping by machine learning improved geometrical optics
calculations. Biomed. Opt. Express.

[8] Liu R., Shao M., Ke Z., Li C., Lu F., Zhong M.-C., Mao Y., Wei X., Zhong Z., Zhou J. (2023). Measurement of red blood cell deformability
during morphological
changes using rotating-glass-plate-based scanning optical tweezers. Biomed. Opt. Express.

[9] Lee K., Danilina A. V., Kinnunen M., Priezzhev A. V., Meglinski I. (2016). Probing the Red Blood Cells Aggregating
Force With
Optical Tweezers. IEEE J. Sel. Top. Quantum
Electron..

[10] Lee K., Kinnunen M., Khokhlova M. D., Lyubin E. V., Priezzhev A. V., Meglinski I., Fedyanin A. A. (2016). Optical tweezers study of red blood
cell aggregation and disaggregation in plasma and protein solutions. J. Biomed. Optt..

[11] Fernandes H. P., Fontes A., Thomaz A., Castro V., Cesar C. L., Barjas-Castro M. L. (2013). Measuring red blood cell aggregation
forces using double
optical tweezers. Scand. J. Clin. Lab. Invest..

[12] Gross, P. ; Farge, G. ; Peterman, E. J. G. ; Wuite, G. J. L. Combining Optical Tweezers, Single-Molecule Fluorescence Microscopy, and Microfluidics for Studies of DNA–Protein Interactions. Methods in Enzymology; Walter, N. G. , Ed.; Academic Press: 2010; Vol. 475, pp 427–453. DOI:10.1016/S0076-6879(10)75017-5. 20627167

[13] Zhu R., Avsievich T., Popov A., Meglinski I. (2020). Optical Tweezers
in Studies of Red Blood Cells. Cells.

[14] Rusciano G., De Luca A. C., Pesce G., Sasso A. (2008). Raman Tweezers as a
Diagnostic Tool of Hemoglobin-Related Blood Disorders. Sensors.

[15] Lin J., Shao L., Qiu S., Huang X., Liu M., Zheng Z., Lin D., Xu Y., Li Z., Lin Y., Chen R., Feng S. (2018). Application
of a near-infrared laser
tweezers Raman spectroscopy system for label-free analysis and differentiation
of diabetic red blood cells. Biomed. Opt. Express.

[16] Bajwa, H. ; Basit, H. Thalassemia. StatPearls Publishing: Treasure Island (FL), 8 August 2023. https://www.statpearls.com/point-of-care/30014. 31424735

[17] Liu S. C., Derick L. H., Zhai S., Palek J. (1991). Uncoupling of the Spectrin-Based
Skeleton from the Lipid Bilayer in Sickled Red Cells. Science.

[18] Peng Z., Li X., Pivkin I. V., Dao M., Karniadakis G. E., Suresh S. (2013). Lipid bilayer and cytoskeletal
interactions in a red
blood cell. Proc. Natl. Acad. Sci. U.S.A..

[19] Shetty S., Bharati S., Chidangil S., Bankapur A. (2021). Optical Trapping and
Micro-Raman Spectroscopy of Functional Red Blood Cells Using Vortex
Beam for Cell Membrane Studies. Anal. Chem..

[20] Jayraj S., Sarmah P., Ghanashyam C., Bankapur A. (2024). Light-sheet Raman tweezers
for whole-cell biochemical analysis of functional red blood cells. Spectrochimica Acta Part A: Molecular and Biomolecular Spectroscopy.

[21] Sarmah P., Ghanashyam C., Khanna R., Bankapur A. (2025). Unraveling biochemical
differences in the membrane of functional RBCs and elliptocytes using
vortex beam-based micro-Raman spectroscopy. Spectrochimica Acta Part A: Molecular and Biomolecular Spectroscopy.

[22] Zhu R., Avsievich T., Su X., Bykov A., Popov A., Meglinski I. (2022). Hemorheological
alterations of red blood cells induced
by 450-nm and 520-nm laser radiation. Journal
of Photochemistry and Photobiology B: Biology.

[23] Chowdhury A., Waghmare D., Dasgupta R., Majumder S. K. (2018). Red blood cell membrane
damage by light-induced thermal gradient under optical trap. Journal of Biophotonics.

[24] Al-Yasiri A. Y. (2018). In Vitro
Influence of Low-Power Diode Laser Irradiation Time on Human Red Blood
Cells. Photomedicine and Laser Surgery.

[25] Brazhe N. A., Brazhe A. R., Sosnovtseva O. V., Abdali S. (2009). Novel chiroptical analysis
of hemoglobin by surface enhanced resonance Raman optical activity
spectroscopy. Chirality.

[26] Brazhe N. A., Abdali S., Brazhe A. R., Luneva O. G., Bryzgalova N. Y., Parshina E. Y., Sosnovtseva O. V., Maksimov G. V. (2009). New Insight into
Erythrocyte through In Vivo Surface-Enhanced Raman Spectroscopy. Biophys. J..

