# Supplementary material for: Toward a Comprehensive Raman Analysis of Functional Cells With Structured Optical Traps
Source: Anal Chem. 2026 Apr 21;98(17):12925–35. doi: 10.1021/acs.analchem.6c00651 (PMC13150814; doi:10.1021/acs.analchem.6c00651)
Supplement: Supplementary file 1 [file ac6c00651_si_001.pdf]

SUPPORTING INFORMATION

Towards a Comprehensive Raman Analysis of Functional Cells With Structured Optical Traps

Panchanil Sarmah[1], Vidya Rastapur[1], Ruchee Khanna[2], Aseefhali Bankapur[,1]*

[1]Manipal Institute of Applied Physics, Manipal Academy of Higher Education, Manipal 576104, India

[2]Department of Pathology, Kasturba Medical College, Manipal, Manipal Academy of Higher Education, Manipal 576104, India

***Corresponding Author**

Email: asif.bankapur@manipal.edu

Address:

Manipal Institute of Applied Physics
LG-01, Academic Block – 5, MIT Campus
Manipal – 576104
Udupi, Karnataka, India

TABLE OF CONTENTS

Sl. No.	Contents	Page no.
1	Figure S1. A schematic of the home-built Raman setup used for the comparison of membrane contribution from point-vortex-line traps	S3
2	Table S1. List of spectral features present and picked up by different PC loadings in point-vortex-line traps	S4 – S13
3	Table S2. P-values of spectral features present and picked up by PC 2 loadings in vortex and line traps. P-values: Welch's ANOVA, Yellow: insignificant	S14
4	References	S15 – S17

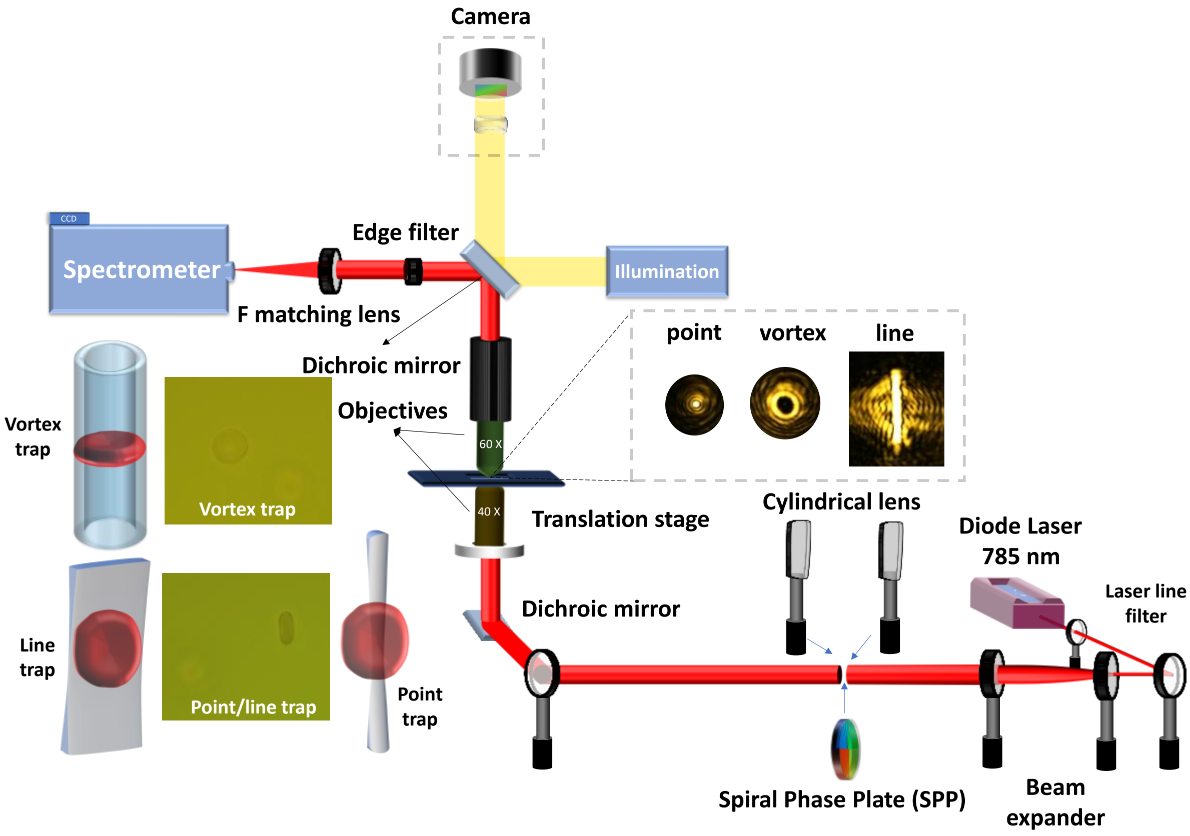

Figure S1. A schematic of the home-built Raman setup used for the comparison of membrane contribution from point-vortex-line traps

Table S1. List of spectral features present and picked up by different PC loadings in point-vortex-line traps

Sl No.	Vortex	Line	Point	Vortex PC2 loadings	Line PC2 loadings	PC1 loadings	Hemoglobin	Protein	Lipid	Carbohydrate
1.	885	---	885	---	---	884	---	Trp [1]	---	Gal-Amine [2]
2.	---	888	---	887	887	---	---	Trp [1]	---	Gal-Amine [2]
3.	889, 893	---	891	893	893	889	---	---	PC [3], PI [3]	
4.	896,899	896, 899	897	897	897	899	---	p: sk: N–C_α–C (α-helix) [4]	---	Sia [5]
5.	906	904	902	907	907	---	---	Lys [6]	PC [3], PS [3]	
6.	913 (s)	913	---	912	---	912	---	---	---	Glu [7]
7.	917	918	---	919	918	---	---	p: sk: N–C_α–C (α-helix) [4]	---	---
8.	927	927	927	---	930	927	---	p: sk: N–C_α–C (α-helix) [4]	---	---
9.	---	931	931 (w)	933	933	---	---	p: sk: N–C_α–C (α-helix) [4], Glut [8]	---	---
10.	935	936	936	935	---	935	---	---	---	---
11.	942(sh)	943	942	941	941	---	ν_{32} [9]	---	---	---
12.	948	949	949	946	946	948	---	---	---	---

13.	952	----	953	---	---	952	---	---	---	---
14.	955	955	----	955	955	---	---	p: sk: N–C_α–C (R Coil) [4]	---	---
15.	962	960	960	---	---	---	---	---	---	---
16.	967	---	967 (hum)	964	964	---	---	p: sk: N–C_α–C (R Coil) [4]	---	---
17.	974	971,973	972,974	973	973	970	ν(C_c-C_d) [9]	---	---	---
18.	981	---	---	980	980	---	---	---	δ(CH) [10]	---
19.	991	991	---	---	---	---	---	Pro [11]	---	---
20.	---	---	993	---	997	995	---	Pro [11]	---	---
21.	1000	1000	1000	---		1001	---	Phe [1]	---	---
22.	1012	----	----	1015	1015	1013	---	Trp [1]	---	---
23.	1018	1017	1017	---	---	---	---	---	---	---
24.	1028	1028	1028	1029	1029	1028	---	Phe [1]	δ(CH) [10]	---
25.	1035	----	1036	1034	1034	1035	---	---	δ(CH) [10]	---
26.	1040	1040	1040	1040	1039	1040	---	---	δ(CH) [10]	---
27.	---	1043	1042	---	---	1043	---	Tyr [1], p: C-N [12]	---	---
28.	1046	1047 (sh)	1047	---	---	1046	---	Arg [6]	---	---

29.	1051	1049	1052	1051, ch cyld	1049	1052	---	Arg [6]	---	Gal [7], Glu [7]
30.	1056	1055	1056	1055	1055	1057	---	---	PS: C-C str [3]	---
31.	1063(sh)	----	1062(sh)	---	---	1062	---	---	SM: C-C str [13]	---
32.	1066	1066	1066	1066	1066	1066	---	Lys [6]	PC [3], PE [3], PI [3], SM [13]	---
33.	---	1071	1070	---	---	1070	---	Hist [14]	---	---
34.	1073	1073	1074	1073	1073	1073	---	Glut [8]	---	Gal [7, 15], Sia [5]
35.	1079	1076	1076	1078	1077	---	---	Trp [1], Lys [6]	---	---
36.	1082	1081	1081	---	---	---	---	PE, CHL: C-C str [3, 10]	---	---
37.	1088	1088	-----	1089	1089	1089	---	Hist [14], Arg [6]	PC, PS, PE : C-C str [3]	Man [7]
38.	1093	----	1092	1094	1094	1093	$\delta(=C_bH_2)_{as}$ [9]	---	PI: Phos Lip: P-O str [13]	---
39.	1113	1113	1113	---	1109	1110	---	p: C-N [16]	SM: C-C str [10]	Fuc [2], Sia [5]
40.	1118	---	---	1118	1118	---	ν_5 [9]	p: C-N [16]	---	---

41.	1124	1123	1123	1124	1124	---	ν_5 [9]	p: C-N [16]	---	---
42.	1130	---	----	---	---	1133	---	p: C-N [16]	SM, CHL: C-C str [10]	Fuc [2]
43.	1142	1142	---	1142	1142	1142	---	---	---	Gal [7], Sia [5]
44.	1148	1148	1147	---	---	---	---	---	---	Glu [7]
45.	1151	1151	1151	1151		1151	ν_{44} [9]	---	---	---
46.	1157	---	---	1157	---	---	---	---	---	Gal [7], Fuc [2], Gal-Amine [2]
47.	---	1161 (sh)	---	---	1160	1161	---	CH_3 rock [17]	---	---
48.	1167	1167	-----	1166	1166		---	Arg [6]	---	---
49.	1171	1170	1169	1172	1172	1171	ν_{30} [9]	---	---	---
50.	1179	---	---	1179	---	1180	---	Arg [6]	---	---
51.	1209	1208	1209	1206,10	---	1206	ν_{5+} ν_{18} [9]	Phe [1]	---	---
52.	1221	1221	1221	1220	---	1221	ν_{13} [9]	---	---	---
53.	1232	1232	---	1232	1232	---	---	Amide III (β-sheet) [4]	---	---
54.	---	1237	1235	---	---	1238	---	Amide III (β-sheet) [4], Hist [14]	---	Gal-Amine [2]

55.	1240	1240	---	1240	1240	---	---	Amide III (β- sheet) [4]	---	---
56.	---	1244	1243	---	---	1244	---	Amide III (Unordered) [4]	---	---
57.	1246	1246	---	1246	1246	1247	---	Amide III (Unordered) [4]	PO_2^- Anti-sym str [18]	Gal [7]
58.	1251	----	1250	---	---	---	---	Amide III (Unordered) [4] Tyr [1]	---	---
59.	1255	1255,57	1257	1255	1255	1257	---	Amide III (β-turn) [4], Trp [1]	---	---
60.	-----	1259	1259		1259	1259	---	Amide III (β-turn) [4], Trp [1]	---	---
61.	1261	1261	1261	1260	1259		---	Amide III (β-turn) [4],	---	---
62.	---	1263	----		1264	1263	---	Amide III (β-turn) [4]	---	---
63.	1267	1267	1267	---	---	---	---	Amide III (β-turn) [4], Tyr, Hist [14]	PC, PS, PI: δ(=CH) [3]	---
64.	1271	1271	1271	1271	1271	---	---	Amide III (α-helix/β-turn) [4]	---	Fuc [2]
65.	1279	--	1277	---	---	1278	---	Amide III (α-helix/β-turn) [4], Trp [1]	---	---
66.	1285	1284	1284	1286	1285	1283	Methylene	---	---	---

							Wag [9]			
67.	1289	1289	1289	1289	1289	---	---	α-helix [4]	---	---
68.	1297	-----	1297	1296	1296	1297	---	---	PS [3], PE [3], SM [10]: $\tau(CH_2)$	---
69.	1299	1302	1301	1301	1301	1302	ν_{21} [9]	---		---
70.	1304	---	---	---	---	---	---	α-helix [4]	PC, PI: $\tau(CH_2)$ [3]	---
71.	1313	1315	1315	---	---	1312	---	Glut [8], p: $\delta(C_\alpha\text{-H})$ [16]	---	---
72.	1325	1324	1324 (hump)	1324	1324	1325	---	Trp [1]	---	Gal [7], Fuc [2], Glu [7]
73.	1336	1335	1335	---	---	---	ν_{41} [9]	$\delta(C_a\text{-H})$ [16]	---	---
74.	1341	1340	1340	1342	---	1343	ν_{41} [9]	---	---	---
75.	-----	1347	----	---	---	---	---	Trp [1]	---	---
76.	1353	---	---	---	---	1352	---	Trp [1]	---	Man [7]
77.	----	1358	----	---	1358	1358	---	Hist [14], Lys [6], Arg [6]	---	---
78.	1367	1367	1366	1367	1367	1367	ν_4 [9]	---	---	---
79.	1372	1372	1372	---	---	---	ν_4 [9]	---	---	Man [7]

80.	1376	---	1375	---	---	1376	ν_4 [9]	---	---	---
81.	1386	1386	1386	---	1384	1384	ν_{12} [9]	---	---	---
82.	1397	1397	1397	1396	---	1397	ν_{40} [9]	---	---	---
83.	1411	1410	1411	---	---	1411	ν_{29} [9]	---	---	---
84.	1417	----	---	1416	1416	1416	---	Lys [6], Arg [6]	---	---
85.	1425	----	---	1425	1425	1425	ν_{28} [9]	---	Lip: β(CH_2) [3]	---
86.	1431	1432	1432	1431	1431	1431	---	---	Lip: β(CH_2) [3]	---
87.	1437	1438	1438	1437	1437	1438	---	Trp [1]		---
88.	---	1441	----	---	1441	-----	---	Hist [14]	Lip: α(CH_2/CH_3) [3]	---
89.	1443	-----	---	1443	---	1443	---	Hist [14]	Lip: α(CH_2/CH_3) [3]	---
90.	1448	1447	1447	1448	1447	1449	---	p: δ(CH_2/CH_3) [16]	---	Fuc [2]
91.	1452	1452	1452	1452	1452	1453	---	p: δ(CH_2/CH_3) [16]	---	---
92.	--	1455	---	1455	1455	---	---	-----	Lip: β(CH_2/CH_3) [3]	---

93.	1458	---	----		1458	1458	---	Glut [8]	---	Man [7], Glu [7] Sia [5]
94.	1461	1460	1460	1463	1465		---	Trp [1]	---	---
95.	1469	1469	1468	1471	1471	1470	---	Lys [6], Arg [6]	---	Ace-Glu [2]
96.	1476	1477	1477	---	---	---	---	p: δ(CH_2/CH_3) [16]	---	---
97.	1481	1479	---	1481	1479	1481	ν_3 [9]	p: δ(CH_2/CH_3) [16]	---	---
98.	1492	---	1492	1493	---	1491	---	His [19]	---	---
99.	1499	---	--	1499	---	---	ν_{39} [9]	---	---	---
100.	1517	---	1520	1514	---	1513	---	Tyr [1]	---	---
101.	1523	1518	1524	1519, 1524	1524	1518, 1525	ν_{38} [9]	---	---	---
102.	1545	1542	1544	1546	1546	1540	ν_{11} [9]	---	---	
103.	1550	---	---	---	---	1550	---	Trp [1]		Ace-Glu [2]
104.	1561	1560	1562	1559	1565	1563	ν_2 [9]	---	---	---
105.	1579	----	---	1577	1577	---	---	Trp [1]	---	---
106.	1582	1579	1581	1577	1577	---	ν_{37} [9]	---	---	---

107.	1601	1605	1603	1602	1602	1607	ν_{10} [9]	---	---	---
108.	1617	1617	1617	1620	1620	1617	$\nu(C_a=C_b)_{vinyl}$ [9]	---	---	---
109.	1626	---	----	---	---	---	---	---	---	Ace-Glu [2]
110.	1635	1635	1635		1635	1634	ν_{10} [9]	---	---	---
111.	1644	----	---	---	---	---	---	Hist [14]	---	Sia [5]
112.	1651	1652	1651	1653	1653	---	---	Amide I (α-helix) [4]	---	---
113.	1656	1656	1656	---	---	---	---	---	Lip: C=C str [3]	---
114.	1662	1663	1662	---	---	---	---	Amide I (Unordered) [4]	---	---
115.	1667	----	---	1667	---	1668	---	Amide I (Unordered) [4]	---	---
116.	1673	----	1673	---	---	1673	---	Amide I (β-sheet) [4]	CHL: C=C str [10, 13]	---
117.	1680	1680	----	---	---	---	---	Amide I (β-sheet) [4]	---	---
118.	1683	1683	----	1683	1683	---	---	Amide I (β-sheet) [4]	---	---
119.	1689	1689	----	1690	1689	---	---	Amide I (β-turn) [4]	---	---
120.	1696	1695	---	1696	1696	1697	---	Amide I (β-turn) [4]	---	---

121.	1701	1701	---	1701	1703	1701	---	Amide I (β-turn) [4]	---	---

α-Scissoring, β-bending, τ-twisting, δ-deformation, Lip-Lipid, PhosLip–Phospholipid, CHL-Cholesterol, Trp-Tryptophan, Tyr-Tyrosine, Phe-Phenylalanine, Hist-Histidine, Arg-Arginine, Lys-Lysine, Pro-Proline, Glut- Glutamic Acid, PC-Phosphatidylcholine, PE-Phosphatidylethanolamine, PS-Phosphatidylserine, PI-Phosphatidylinositol, SM-Sphingomyelin, Gal-galactose, Man-mannose, Glu-glucose, Fuc-fucose, and Sia-sialic, (Ace-Glu)-(acid N-acetyl-D-glucosamine) and, (Ace-Gal)-(N- acetyl-D-galactosamine).

Table S2. P-values of spectral features present and picked up by PC 2 loadings in vortex and line traps. P-values: Welch's ANOVA, Yellow: insignificant

Sl. No.	Raman peak	Vortex trap	Line trap	Sl. No.	Raman peak	Vortex trap	Line trap
1	887	< .001	< .001	29	1260	< .001	< .001
2	893	0.001	< .001	30	1271	< .001	< .001
3	902	0.017	< .001	31	1286	< .001	< .001
4	907	0.094	0.055	32	1289	< .001	< .001
5	919	< .001	0.35	33	1296	0.204	< .001
6	955	< .001	0.001	34	1301	< .001	< .001
7	964	0.004	< .001	35	1324	< .001	< .001
8	973	0.006	< .001	36	1367	< .001	< .001
9	980	< .001	0.008	37	1416	< .001	0.086
10	1015	< .001	< .001	38	1431	< .001	< .001
11	1029	< .001	< .001	39	1437	< .001	< .001
12	1034	< .001	0.186	40	1452	< .001	< .001
13	1040	< .001	0.096	41	1458	< .001	< .001
14	1055	< .001	0.085	42	1463	< .001	< .001
15	1066	< .001	< .001	43	1471	< .001	< .001
16	1073	< .001	< .001	44	1481	< .001	0.023
17	1078	0.003	< .001	45	1519	< .001	< .001
18	1089	< .001	0.14	46	1524	< .001	< .001
19	1094	< .001	0.023	47	1546	< .001	< .001
20	1118	< .001	< .001	48	1559	< .001	0.82
21	1124	0.017	< .001	49	1577	< .001	< .001
22	1142	< .001	0.012	50	1602	< .001	< .001
23	1166	< .001	0.001	51	1620	< .001	0.237
24	1172	< .001	0.641	52	1653	< .001	< .001
25	1232	< .001	< .001	53	1683	< .001	< .001
26	1240	< .001	< .001	54	1690	< .001	< .001
27	1246	< .001	< .001	55	1696	< .001	< .001
28	1255	< .001	< .001	56	1701	< .001	0.061

References

1. Hernández, B.; Pflüger, F.; Adenier, A.; Kruglik, S. G.; Ghomi, M., Vibrational Analysis of Amino Acids and Short Peptides in Hydrated Media. VIII. Amino Acids with Aromatic Side Chains: l-Phenylalanine, l-Tyrosine, and l-Tryptophan. *The Journal of Physical Chemistry B* **2010,** *114* (46), 15319-15330. DOI: https://doi.org/10.1021/jp106786j

2. De Gelder, J.; De Gussem, K.; Vandenabeele, P.; Moens, L., Reference database of Raman spectra of biological molecules. *Journal of Raman Spectroscopy* **2007,** *38* (9), 1133-1147. DOI: https://doi.org/10.1002/jrs.1734

3. Pezzotti, G.; Horiguchi, S.; Boschetto, F.; Adachi, T.; Marin, E.; Zhu, W.; Yamamoto, T.; Kanamura, N.; Ohgitani, E.; Mazda, O., Raman Imaging of Individual Membrane Lipids and Deoxynucleoside Triphosphates in Living Neuronal Cells during Neurite Outgrowth. *ACS Chemical Neuroscience* **2018,** *9* (12), 3038-3048. DOI: https://doi.org/10.1021/acschemneuro.8b00235

4. Di Foggia, M.; Taddei, P.; Torreggiani, A.; Dettin, M.; Tinti, A., Self-assembling peptides for biomedical applications: IR and Raman spectroscopies for the study of secondary structure. *Proteomics Research Journal* **2011,** *2* (3), 231.

5. Vinogradova, E.; Tlahuice-Flores, A.; Velazquez-Salazar, J. J.; Larios-Rodriguez, E.; Jose-Yacaman, M., Surface-enhanced Raman scattering of N-acetylneuraminic acid on silver nanoparticle surface. *Journal of Raman Spectroscopy* **2014,** *45* (9), 730-735. DOI: https://doi.org/10.1002/jrs.4544

6. Hernández, B.; Pflüger, F.; Derbel, N.; De Coninck, J.; Ghomi, M., Vibrational Analysis of Amino Acids and Short Peptides in Hydrated Media. VI. Amino Acids with Positively Charged Side Chains: l-Lysine and l-Arginine. *The Journal of Physical Chemistry B* **2010,** *114* (2), 1077-1088. DOI: https://doi.org/10.1021/jp909517r

7. Wells, H. A.; Atalla, R. H., An investigation of the vibrational spectra of glucose, galactose and mannose. *Journal of Molecular Structure* **1990,** *224*, 385-424. DOI: https://doi.org/10.1016/0022-2860(90)87031-R

8. Navarrete, J. T. L.; Hernández, V.; Ramírez, F. J., Vibrational study of aspartic acid and glutamic acid dipeptides. *Journal of Molecular Structure* **1995,** *348*, 249-252. DOI: https://doi.org/10.1016/0022-2860(95)08635-9

9. Hu, S.; Smith, K. M.; Spiro, T. G., Assignment of Protoheme Resonance Raman Spectrum by Heme Labeling in Myoglobin. *Journal of the American Chemical Society* **1996,** *118* (50), 12638-12646. DOI: https://doi.org/10.1021/ja962239e

10. Czamara, K.; Majzner, K.; Pacia, M. Z.; Kochan, K.; Kaczor, A.; Baranska, M., Raman spectroscopy of lipids: a review. *Journal of Raman Spectroscopy* **2015,** *46* (1), 4-20. DOI: https://doi.org/10.1002/jrs.4607

11. Zhu, G.; Zhu, X.; Fan, Q.; Wan, X., Raman spectra of amino acids and their aqueous solutions. *Spectrochimica Acta Part A: Molecular and Biomolecular Spectroscopy* **2011,** *78* (3), 1187-1195. DOI: https://doi.org/10.1016/j.saa.2010.12.079

12. Ghanashyam, C.; Shetty, S.; Bharati, S.; Chidangil, S.; Bankapur, A., Optical Trapping and Micro-Raman Spectroscopy of Functional Red Blood Cells Using Vortex Beam for Cell Membrane Studies. *Analytical Chemistry* **2021,** *93* (13), 5484-5493. DOI: https://doi.org/10.1021/acs.analchem.0c05204

13. Krafft, C.; Neudert, L.; Simat, T.; Salzer, R., Near infrared Raman spectra of human brain lipids. *Spectrochimica Acta Part A: Molecular and Biomolecular Spectroscopy* **2005,** *61* (7), 1529-1535. DOI: https://doi.org/10.1016/j.saa.2004.11.017

14. Pflüger, F.; Hernández, B.; Ghomi, M., Vibrational Analysis of Amino Acids and Short Peptides in Hydrated Media. VII. Energy Landscapes, Energetic and Geometrical Features of L-Histidine with Protonated and Neutral Side Chains. *The Journal of Physical Chemistry B* **2010,** *114* (27), 9072-9083. DOI: https://doi.org/10.1021/jp103348y

15. Wiercigroch, E.; Szafraniec, E.; Czamara, K.; Pacia, M. Z.; Majzner, K.; Kochan, K.; Kaczor, A.; Baranska, M.; Malek, K., Raman and infrared spectroscopy of carbohydrates: A review. *Spectrochimica Acta Part A: Molecular and Biomolecular Spectroscopy* **2017,** *185*, 317-335. DOI: https://doi.org/10.1016/j.saa.2017.05.045

16. Rygula, A.; Majzner, K.; Marzec, K. M.; Kaczor, A.; Pilarczyk, M.; Baranska, M., Raman spectroscopy of proteins: a review. *Journal of Raman Spectroscopy* **2013,** *44* (8), 1061-1076. DOI: https://doi.org/10.1002/jrs.4335

17. Milanovich, F. P.; Shore, B.; Harney, R. C.; Tu, A. T., Raman spectroscopic analysis of Dutch Belt rabbit erythrocyte ghosts. *Chemistry and Physics of Lipids* **1976,** *17* (1), 79-84. DOI: https://doi.org/10.1016/0009-3084(76)90038-4

18. Mushayakarara, E.; Albon, N.; Levin, I. W., Effect of water on the molecular structure of a phosphatidylcholine hydrate: Raman spectroscopic analysis of the phosphate, carbonyl and carbon-hydrogen stretching mode regions of 1,2-dipalmitoylphosphatidylcholine dihydrate. *Biochimica et Biophysica Acta (BBA) - Biomembranes* **1982,** *686* (2), 153-159. DOI: https://doi.org/10.1016/0005-2736(82)90107-9

19. Takeuchi, H., Raman structural markers of tryptophan and histidine side chains in proteins. *Biopolymers* **2003,** *72* (5), 305-317. DOI: https://doi.org/10.1002/bip.10440
